# Psychometric Properties of the Norwegian Version of the Cognitive Therapy Adherence and Competence Scale (CTACS) and Its Associations With Outcomes Following Treatment in IAPT Norway

**DOI:** 10.3389/fpsyg.2021.639225

**Published:** 2021-02-16

**Authors:** Linn Vathne Lervik, Marit Knapstad, Asle Hoffart, Otto R. F. Smith

**Affiliations:** ^1^Division of Mental and Physical Health, Department of Health Promotion, Norwegian Institute of Public Health, Bergen, Norway; ^2^Department of Clinical Psychology, University of Bergen, Bergen, Norway; ^3^Department of Psychology, University of Oslo, Oslo, Norway; ^4^Modum Bad Psychiatric Center, Vikersund, Norway

**Keywords:** CBT, CTACS, IAPT Norway, competence, alliance, psychometric properties, associations with outcome

## Abstract

**Background:** No studies have examined the underlying structure or predictive validity of the Cognitive Therapy Adherence and Competence Scale (CTACS). Examining the structure of the CTACS is of great relevance because it could provide information on what constitutes competence in CBT, and whether some underlying factors are more important for predicting treatment outcomes than others. This study investigates the psychometric properties of the Norwegian version of CTACS and its associations with treatment outcomes in a sample of primary care clients who received CBT for anxiety and/or depression.

**Method:** Independent assessors rated audiotaped therapy sessions (early, mid and late in treatment) in a sample of 132 primary care clients (mean [SD] age = 34.8 [11.8], 63.6% women), participating in the Prompt Mental Health Care trial. Outcomes were symptoms of anxiety and depression assessed by patient self-report questionnaires. Structural validity was examined by means of confirmatory and exploratory factor analyses (CFA/EFA), whereas longitudinal associations with treatment outcome were explored by adopting multilevel modeling.

**Results:** No evidence was found for the divergent validity of the constructs competence and adherence as indicated by a very high correlation between these two subscales in CTACS (0.97). Regarding reliability, ICCs for the mean score of the full competence scale and its associated subscales were generally good to excellent (0.70–0.80), although the subscale measuring the quality of the therapeutic relationship was relatively low (0.44). Internal consistency was overall acceptable, but our CFA models did not provide an acceptable fit for the pre-specified one-factor and four-factor solutions. EFA results were difficult to interpret, with a sub-optimal three-factor solution providing best model fit and only two meaningful factors [CBT specific skills (α = 0.82) and session structure (α = 0.59)]. Overall, the results indicated no evidence for the scales' predictive validity.

**Conclusion:** Our findings point to several psychometric problems of the CTACS that may limit both its research and clinical utility. The importance of providing empirical evidence for both reliability and validity aspects of scales are discussed and suggestions for future research are provided.

## Introduction

The British Improving Access to Psychological Therapies (IAPT) and the Norwegian adaptation Prompt Mental Health Care (PMHC; in Norwegian RPH) are relatively new treatment models, widely implemented to address the treatment gap for common mental health disorders such as anxiety and depression. Both approaches have been positively evaluated (Clark et al., 2009; Knapstad et al., 2018), and evidence for the effectiveness of PMHC was recently provided by means of a randomized controlled trial (Knapstad et al., 2020). Cognitive Behavioral Therapy (CBT) is the respective initiatives' main methodology, and even though CBT is documented to be effective (Hofmann and Smits, 2008; Olatunji et al., 2010), there is much uncertainty and debate about the active ingredients that can explain its effectiveness.

Treatment fidelity and alliance are arguably the two factors that have been studied most. The former concerns adherence - the degree to which therapists are delivering the theory-specified techniques or methods of the intervention- and competence–the skill with which these techniques and methods are delivered (Perepletchikova, 2011). The latter can be defined as the overall collaborative facets of the client-therapist relationship (Hatcher and Barends, 2006; Flückiger et al., 2018). Based on the current literature, there is some evidence that a good working alliance is associated with better outcomes in CBT (Flückiger et al., 2018), while this association is more uncertain and likely to be weaker for treatment fidelity (Webb et al., 2010).

Much of the work done on these factors is hampered by a range of methodological limitations (Perepletchikova and Kazdin, 2005; Elliott, 2010; Webb et al., 2010; Cuijpers et al., 2019). One major challenge is that the constructs of treatment fidelity and alliance are complex to define and even harder to measure (Barber et al., 2007; Ardito and Rabellino, 2011; Muse and McManus, 2013; Flückiger et al., 2018; Kühne et al., 2020). This seems also reflected in the great variety of measurement scales that are being used (Barber et al., 2007; Ardito and Rabellino, 2011; Muse and McManus, 2013; Goldberg et al., 2020; Kühne et al., 2020), both in terms of actual measures, but also in terms of their modes of administration and assessment (i.e., audio vs. video/client, therapist, vs. expert rated). Moreover, efforts to systemically examine the measurement properties of the available instruments seem to be limited so far (Goldberg et al., 2020).

An instrument that is often used in Norway in both training and research is the Cognitive Therapy Adherence and Competence Scale (CTACS) (Barber et al., 2003). This measure was partly based on the Cognitive Therapy Scale (CTS) (Young and Beck, 1980) and developed to provide overall scores for adherence and competence. Each of the 25 items is rated simultaneously for both adherence (scale ranging from 0 “none” to 6 “thorough”) and competence (scale ranging from 0 “poor” to 6 “excellent”) (Barber et al., 2003). The scale itself consists of four sections and an additional overall performance item: CT structure (nine items), development of a collaborative therapeutic relationship (6 items), development and application of the case conceptualization (six items), and cognitive and behavioral techniques (three items).

Acceptable interrater reliability has been shown for the majority of CTACS items, and internal consistency was high for both the adherence and competence scale (Barber et al., 2003). Evidence for criterion validity was found by showing that CTACS was able to differentiate between different therapeutic modalities (Barber et al., 2003). Several empirical reports have indicated though that it may be difficult to distinguish between adherence and competence and overall ratings are often used (Barber et al., 2003, 2006; Bjaastad et al., 2016; Haug et al., 2016).

To our knowledge, no studies have examined the underlying structure of the CTACS. However, examining the structure of the CTACS is of great relevance because it could provide information on what constitutes competence in CBT, and whether some underlying factors are more important for predicting treatment outcomes than others. Some explanatory work has already been done on the CTS (Vallis et al., 1986; Affrunti and Creed, 2019; Goldberg et al., 2020) and on a heavily modified version of CTACS (Bjaastad et al., 2016). The former found both a two-factor (CBT skill and General therapy skill) and three factors (structure, CBT techniques, and therapeutic relationship skills), while the latter identified two factors (CBT structure and session goals, and process and relational skills).

The primary aims of the current study were therefore to examine the psychometric properties of the CTACS (including its structural validity), and its associations with treatment outcome in a sample of Norwegian primary care clients who received CBT for anxiety and/or depression.

## Methods

### Setting

This study used data from the treatment arm of the PMHC study in two municipalities in South-West Norway, enrolling participants from November 2015 to August 2017. Key characteristics of the PMHC service is to offer short term, free of charge, stepped care approach with no need for referrals, and providing CBT from interdisciplinary teams. Details regarding the trial can be found elsewhere, ClinicalTrials.gov Identifier: NCT03238872 (Knapstad et al., 2020).

Both PMHC teams consisted of four full-time therapist equivalents, including a clinical psychologist in at least 50% position. All therapists had individual treatment responsibilities, however, the clinical psychologist had the overall professional responsibility. Three therapists in Kristiansand quitted during the trial period and were not replaced.

All the PMHC therapists needed to have at least three years of relevant higher education and most of them were trained nurses, social workers and social educators. Also, they completed mandatory one-year training in CBT. The training was inspired by the IAPT curriculum albeit adjusted to Norwegian conditions. The treatment offered included both low intensity (guided self-help and group-based psychoeducation) and high intensity (face-to-face individual therapy) treatment forms (Knapstad et al., 2020).

### Procedures

All clients contacting PMHC were offered to participate in a first assessment session. Here instructions about the study and treatment methodology were provided, and the therapist assessed relevant information to decide whether PMHC could be the appropriate treatment or not. The therapist identified the relevance and severity of the mental problems and available client resources. Participation was based on opt-in, where clients who were suitable for treatment were invited to participate and asked to sign an informed consent. A full description of in- and exclusion criteria is provided elsewhere (Knapstad et al., 2020). Clients completed questionnaires during the initial assessment, and before each session during the treatment. The study was approved by the Regional Ethics Committee for Western Norway (REK-vest no. 2015/885).

### Participants and Data Material

#### Clients

The total number of participants in the PMHC group in the RCT trial was 526. However, only those who received at least four individual face-to-face sessions were included in the analysis for the current study (*N* = 132). Of these, 37.9% (*n* = 50) were rated by the therapists as having primarily symptoms of depression, 28.0% (*n* = 37) had primarily symptoms of anxiety, and 34.1% (*n* = 45) had both symptoms of anxiety and depression.

#### Therapists

Ten therapists from the two sites provided the PMHC treatment. Each therapist treated on average 13 clients (range: 1–31 clients). Note that without the one therapist who only treated one client, the range would be from 6-31 clients. Seventy percent (*n* = 7) of the therapists were females, and 30% (*n* = 3) were clinical psychologists.

#### Materials

**Figure 1:**
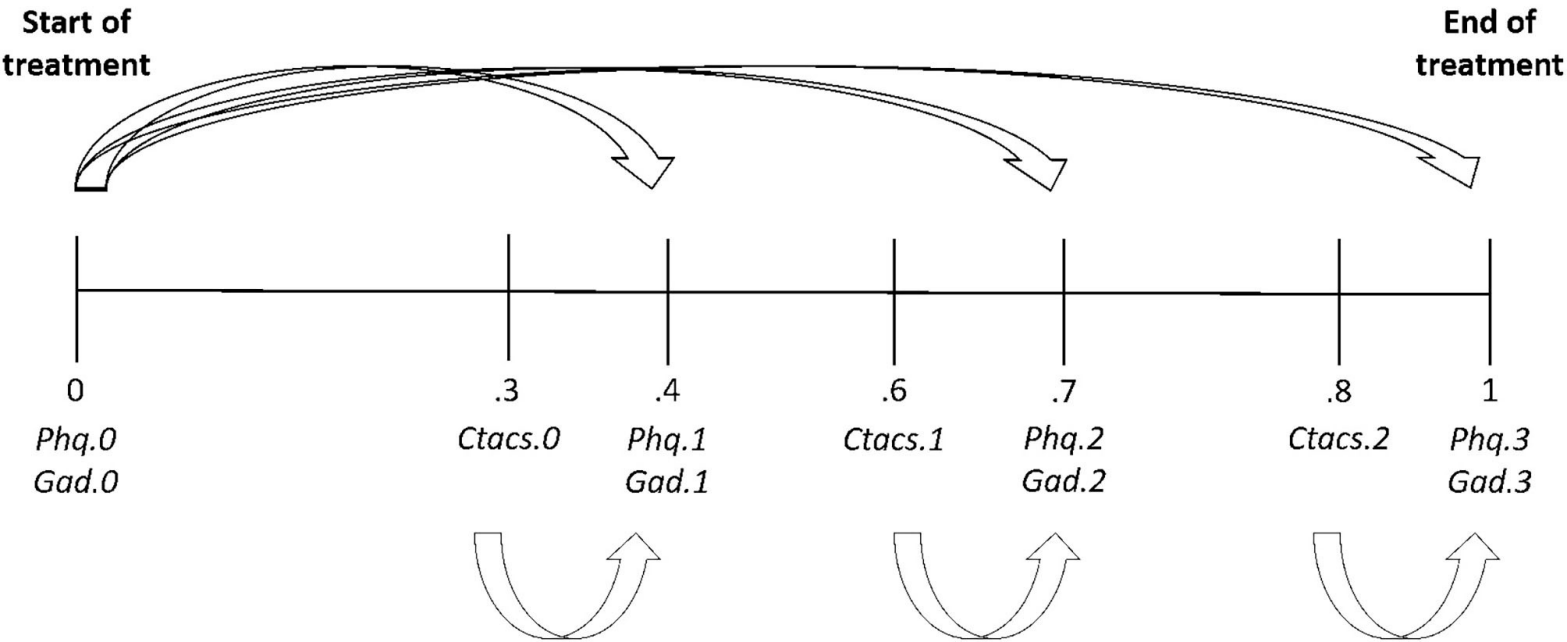
Generic path diagram displaying the average relative positions of measurement occasions in the course of treatment and the proposed associations between competence and therapy outcome variables.

Upon availability, three audiotapes were selected for each included client. One session from early (106 tapes), mid (114 tapes), and toward the end (113 tapes) of the treatment period. Audiotapes were available at only one time point for 8.4% of clients (*n* = 7), at two time points for 37.1% (*n* = 49) and at all three time points for 57.6% (*n* = 76). First and final sessions were not included to avoid confounding with assessment and outcome. The PMHC therapists did not know which sessions would be selected for evaluation. As number and timing of sessions were not standardized in PMHC, audiotapes and outcomes scores were selected based on their relative position in the course of therapy with an average relative time of approximately.3,0.6 and.8 for audio recordings (see Figure 1).

#### Treatment

The included 132 clients were treated with a median of 10 (IQR 6.5) CBT sessions. Notably, consistent with the PMHC treatment model some clients received a combination of treatment types. In this study sample, 71 (54%) of the clients received primarily individual face-to-face treatment and 61 (46%) of the clients received a combination of low- (guided self-help or group-based psychoeducation) and high-intensity (face-to-face) treatment. As a result of the target group having symptoms related to several anxiety disorders and depression, the provided treatments consisted of multiple specific CBT treatment protocols. Example of provided protocols was the Clark and Wells treatment manual for social anxiety (Wells, 1997; Clark et al., 2005), Clark and Salkovski treatment manual for panic-disorder (Hawton et al., 1989), Beck depression model (Beck, 1979) as well as Wells Metacognitive model for rumination and worry (Wells, 2006).

### Fidelity measures

#### The Cognitive Therapy Adherence and Competence Scale (CTACS)

This is an observer-rated scale consisting of 25 items addressing CBT processes or interventions such as *homework* and *guided discovery*. Each item is rated simultaneously for both adherence (scale ranging from 0 “none” to 6 “thorough”) and competence (scale ranging from 0 “poor” to 6 “excellent”) (Barber et al., 2003). For competence assessment, additional descriptions are provided for even numbers (i.e., 0 “the therapist seems unaware of the patients agenda” and 6 “the therapist set an excellent agenda…”).The scale is divided into four domains consisting of CBT structure, development of a collaborative therapeutic relationship, development and application of the case conceptualization, and cognitive and behavioral techniques. There is also a separate assessment for the client's degree of difficulty (one item). In CTACS, adherence is described as the degree to which the therapist engages in the process or intervention. Competence has been equated with quality and appropriateness and described as how well the intervention or process was performed. As described in the introduction, it has been proven difficult to separate adherence and competence empirically (Barber et al., 2003). In our dataset, the correlation between the mean adherence score and the mean competence score was also very strong (*r* = 0.97), suggesting lack of divergent validity. Results of the present study were therefore based on the competence items only. Adequate fidelity was defined as a mean CTACS score >3.0 (Haug et al., 2016). Two items evaluated as not applicable in the PMHC context were excluded; item 17 (“Eliciting core beliefs and schemas”) and item 20 (“Case conceptualization: Linking past to present”).

### Outcome Measures

#### The Patient Health Questionnaire-9 (PHQ-9)

The PHQ-9 is a self-report instrument consisting of nine items covering the DSM-IV criteria for major depression. The items are scored for the last two weeks on a four-point scale ranging from 0 (not at all) to 3 (almost every day) with total scores ranging from 0 to 27. Example items are “Feeling bad about yourself – or that you are a failure or have let yourself or your family down” and “little interest or pleasure in doing things” (Kroenke et al., 2001). The PHQ-9 has demonstrated good psychometric properties (Kroenke et al., 2001, 2010). In the trial sample, Cronbach's alpha for the instrument was 0.80.

#### The Generalized Anxiety Disorder 7-Item Scale (GAD-7)

The GAD-7 is a self-report instrument consisting of seven items covering the DSM-IV criteria for generalized anxiety disorder. The items are scored on the same scale (0 “not at all” to 3 “nearly every day”) and duration (two weeks) as for PHQ-9. Sum scores ranging from 0 to 21. Example items are “Worrying to much about different things” and “feeling afraid as if something awful might happen” (Spitzer et al., 2006; Kroenke et al., 2010). GAD-7 has shown satisfactory sensitivity and specificity for several anxiety disorders (Kroenke et al., 2007, 2010). Cronbach's alpha from the trial sample was 0.83.

### Audiotape Assessment

Before recruiting and training of student raters, the expert rater of this study, LVL, calibrated her ratings with an expert in the field and co-author of the study, AH. Three audiotaped recordings were independently rated, deviant scores were discussed and the final score agreed upon. Four senior clinical psychology students were recruited. All received a three days training course by LVL followed by independent ratings of 10 training sessions. One student got sufficient training scores (ICC = 0.89 for total CTACS score) and was selected to rate the audiotaped sessions for the study. The expert rater is a clinical psychologist with a two years CBT education, specialization in clinical work psychology, and has several years of clinical experience particularly working with anxiety and depression and CBT methodology. None of the evaluators of the audiotaped therapy sessions were involved in providing treatment and both were blind to the outcome for the clients in this study. The expert rater analyzed 217 tapes, whereas the student rater analyzed 145 tapes. Twenty-nine sessions were rated by both raters. For these, intraclass correlations [ICC(3,1)] were calculated based on a two-way mixed-effects model (consistency, single rater/measurement). Cicchetti's (1994) guidelines (Cicchetti, 1994) were used to interpret the ICCs (lower than 0.40 = poor, between 0.40 to 0.59 = fair, between 0.60 and 0.75 = good, higher than 0.75 = excellent). ICCs were calculated for mean (sub)scale scores and individual items.

### Statistical Analysis

Descriptive statistics were calculated for baseline, process and outcome variables. Chi-square tests for categorical variables and independent samples t-tests for continuous variables were used to compare the sample of the present study (*N* = 132) with clients from the original sample who were not included (*N* = 394). Alpha level was set to.05 for all models. The no-change hypothesis was tested for CTACS, PHQ-9, and GAD-7 by means of linear mixed models with time point as factor variable (1–3 for CTACS, 1–4 for PHQ-9/GAD-7). Effect size (ES) estimates were calculated by having the first and last measurement in the nominator and the standard deviation of the first measurement as the denominator.

All subsequent models were estimated using full information maximum likelihood estimation under the assumption of data missing at random with robust standard errors (maximum likelihood with robust standard errors).

To test the proposed underlying structure of CTACS, a 1- and 4-factor confirmatory factor analysis models were tested across all sessions (*N* = 333). Dependency between sessions was accounted for by using the type = complex feature in Mplus. Model fit was assessed by using the comparative fit index (CFI), the root mean square error of approximation (RMSEA), and the standardized root mean square residual (SRMR). A CFI of.95 or greater, RMSEA of.06 or less, and SRMR of 0.08 or less were considered indicative of good model fit (Chen, 2007).

Longitudinal multilevel models were used to examine the predictive validity of CTACS by exploring whether variability in competence was associated with subsequent symptom levels of anxiety or depression. Measurement occasions (level 1) were nested within clients (level 2). Person mean centering of the competence (sub)scales was used to separate within- and between-person effects. The within-person effect is here represented by the session-specific deviation from the client-specific average scores. The between-person effect is represented by the client-specific average of the respective competence (sub)scales throughout treatment (i.e., the person mean). Due to the relatively small number of therapists in this study, the therapist variance was modeled by adding it as a fixed effect at level 2. For estimation purposes, the therapist with only one client was excluded from these analyses. A visual check was performed to determine whether sufficient variance in competence scores remained after partialling out therapist effects (see supplementary file in Supplementary Material). Baseline levels of anxiety/depression were added as a covariate and its effect was allowed to vary across measurement occasions (Figure 1). Initial models assumed time-invariant effects of the competence (sub)scales. In subsequent models, these restrictions were relaxed and evaluated by means of Wald tests. We also checked whether the expert/student rated difficulty of the client affected the observed associations between competence and therapy outcome scores. Stata version 15 and Mplus version 8.2 were used for data analyses.

## Results

### Descriptive Statistics

#### Sample Characteristics

Clients' mean symptom score at baseline was 13.74 (SD = 5.09, range 0–27) for depression and 11.67 (SD = 4.55, range 0–21) for anxiety.This is in line with the intended target group of PMHC and IAPT, although the latter seems to include clients with somewhat higher case severity at baseline (Clark et al., 2009; Knapstad et al., 2020). Of the 132 clients included in this study, 63.6% (*n* = 84) were women. The average age was 34.8 years (SD = 11.8), and 38.5% (*n* = 50) of the participants did not have a partner. Concerning educational level, 8.3% (*n* = 11) of the sample had primary education only, 37.9% (*n* = 50) had a high school education, whereas 52.3% (*n* = 69) had higher education. The percentage of participants with an immigrant background was 7.6% (*n* = 10). Finally, 37.9% (*n* = 50) was in regular work at baseline, 40.2% (*n* = 53) in combined work and recipients of benefits (fully or graded sick leave or graded work assessment allowance/disability benefits), and 22.0% (*n* = 29) was out of work with or without benefits.

#### Comparison of Study Sample With Excluded Participants

No significant discrepancies were found regarding age, gender, educational level, civil status, employment, immigrant background or symptom severity of anxiety and depression at baseline. As expected, they did significantly differ concerning treatment type and the number of sessions. The current sample had a significantly higher average amount of sessions (10.9 vs. 4.6, *p* = 0.001). The current sample was also much more likely to primarily receive high-intensity face-to-face treatment (53.2 vs. 20.2%) or a combination of low- and high-intensity treatment (46.2 vs. 27.1%) as compared to clients who were excluded (*p* < 0.001).

#### Changes Over Time

Overall, the sessions mean competence scores were close to, but below, the predefined level (>3.0) for adequate competence on the CTACS scale across measurement occasions (occasion 1: M = 2.75, SD = 0.74, range = 3.36; occasion 2: M = 2.62, SD = 0.78, range = 3.23; occasion 3: M = 2.52, SD = 0.70, range = 2.86). The observed differences across measurement occasions were approaching statistical significance [F_(2,202.9)_ = 14.6, *p* < 0.001; ES = 0.31]. A statistically significant decline in competence scores over time was observed across all 4 subscales with effect sizes ranging from.14 (alliance subscale) to 0.36 (structure subscale). PHQ-9 and GAD-7 scores decreased between the first and fourth measurement occasions with an average change of 7.2 for PHQ-9 (*p* < 0.001; ES = 1.41), and an average change of 6.4 for GAD-7 (*p* < 0.001; ES = 1.39).

### Measurement Properties

#### Reliability

ICCs for the mean score of the full competence scale and its associated subscales were generally good to excellent, although the subscale measuring quality of the therapeutic relationship was relatively low (see Table 1). On the item-level, several items displayed low agreement between raters. In particular items related to therapeutic relationship had low ICCs (items 11–14). Internal consistency was acceptable for both the full scale (α = 0.91), and subscales therapy structure (α = 0.76), therapeutic relationship (α = 0.84), and CBT techniques (α = 0.73). For the subscale case conceptualization, Cronbach's alpha was only 0.61.

**Table 1:** Means, SD and ICC reliability coefficients for the competence items of CTACS.

**Item no**.	**Item description**	**Overall mean (SD) (*n* = 333)**	**Rater 1 mean (SD) (*n* = 217)**	**Rater 2 mean (SD) (*n* = 145)**	**Consistency ICC (3,1) (*n* = 29)**
1	Agenda	0.96 (1.28)	0.96 (1.29)	0.94 (1.27)	0.55
*2*	*Mood check*	*2.37 (1.51)*	*1.98 (1.46)*	*3.05 (1.38)*	*0.25*
3	Bridge from previous visit	1.94 (1.50)	2.27 (1.45)	1.46 (1.50)	0.65
4	Inquired about ongoing problem	3.74 (0.86)	3.65 (0.78)	3.94 (0.96)	0.50
5	Reviewing previous homework	1.91 (1.72)	2.15 (1.75)	1.57 (1.62)	0.60
6	Assigning new homework	2.17 (1.43)	2.32 (1.43)	1.99 (1.44)	0.72
*7*	*Capsule summaries*	*3.00 (1.23)*	*3.01 (1.43)*	*3.03 (0.82)*	*0.12*
*8*	*Patient summary and feedback*	*2.73 (1.05)*	*2.83 (1.05)*	*2.63 (1.08)*	*0.30*
9	Focus/structure	3.16 (1.27)	2.51 (1.07)	4.19 (0.83)	0.58
10	Socialization to CBT model	3.09 (1.33)	3.05 (1.32)	3.23 (1.31)	0.60
*11*	*Warmth/ genuineness/ congruence*	*4.16 (0.77)*	*3.74 (0.64)*	*4.83 (0.47)*	*0.16*
*12*	*Acceptance/Respect*	*4.11 (0.98)*	*3.52 (0.68)*	*5.04 (0.70)*	*0.28*
*13*	*Attentiveness*	*3.15 (0.67)*	*3.09 (0.49)*	*3.31 (0.89)*	*0.28*
*14*	*Accurate empathy*	*3.53 (0.78)*	*3.33 (0.78)*	*3.85 (0.67)*	*0.19*
15	Collaboration	2.93 (1.21)	2.59 (1.11)	3.55 (1.13)	0.45
16	Eliciting automatic thoughts	2.65 (1.41)	2.49 (1.55)	2.96 (1.18)	0.44
18	Eliciting meaning/ understanding/ attributions	2.38 (1.35)	2.22 (1.54)	2.68 (1.00)	0.54
*19*	*Addressing key issues*	*2.59 (1.80)*	*1.54 (1.64)*	*4.19 (0.60)*	*0.29*
21	Sharing conceptualization with patient	1.31 (1.61)	1.60 (1.75)	0.93 (1.37)	0.47
22	Guided discovery	2.66 (1.24)	2.38 (1.39)	3.13 (0.89)	0.55
23	Asking for evidence/alternative views	1.70 (1.49)	1.38 (1.54)	2.19 (1.33)	0.57
24	Use of alternative CBT techniques	1.46 (1.63)	1.13 (1.53)	2.01 (1.71)	0.61
25	Overall performance rating	2.40 (1.14)	2.45 (1.16)	2.36 (1.13)	0.73
	Competence mean score (all items)	2.63 (0.75)	2.45 (0.72)	2.95 (0.69)	0.80
	Therapy structure Mean score (items 1–9)	2.45 (0.78)	2.42 (0.81)	2.54 (0.73)	0.75
	Therapeutic relationship mean score (items 10–15)	3.49 (0.74)	3.22 (0.63)	3.96 (0.68)	0.44
	Case conceptualization mean score (items 16–21)	2.24 (1.05)	1.96 (1.12)	2.69 (0.84)	0.70
	CBT techniques (items 22–24)	1.94 (1.18)	1.63 (1.18)	2.44 (1.06)	0.79

*Items with ICC ≤ .30 appear in italic*.

#### Structural Validity

As shown in Table 2, both the one-factor and the four-factor CFA model did not provide acceptable model fit (CFI = 0.661, RMSEA = 0.122 SRMR = 0.103 and CFI = 0.712, RMSEA = 0.115, SRMR = 0.102, respectively). Modification indices indicated that many items contributed to poor model fit, and not just a small set of items. As interrater reliability was low for several items, we conducted exploratory factor analyses (EFA) (1–4 factors) in which only items with an ICC>0.40 were included (14 of 22 items). The Bayesian information criteria (BIC) suggested that a 3-factor model (BIC = 14376) fitted best as compared to 1- (BIC = 14575), 2- (BIC = 14433), and 4-factor (BIC = 14406) models. Only two of the three factors were interpretable with one factor concerning CBT specific skills (α = 0.82), and another factor concerning session structure (α = 0.59, see also Table 3). We considered the two-factor model as well, but this solution was less interpretable.

**Table 2:** CFA for CTACS based on one-factor and a four-factor solutions, standardized (*n* = 333).

**Item no**.	**Item description**	**1-factor model**	**4-factor model**			
		**F1**	**F1**	**F2**	**F3**	**F4**
1	Agenda	0.397	0.407			
*2*	*Mood check*	0.578	0.589			
3	Bridge from previous visit	0.338	0.334			
4	Inquired about ongoing problem	0.649	0.653			
5	Reviewing previous homework	0.230	0.221			
6	Assigning new homework	0.379	0.367			
*7*	*Capsule summaries*	0.445	0.472			
*8*	*Patient summary and feedback*	0.599	0.606			
9	Focus/structure	0.800	0.818			
10	Socialization to CBT model	0.734		0.700		
*11*	*Warmth/ genuineness/ congruence*	0.614		0.642		
*12*	*Acceptance/Respect*	0.711		0.747		
*13*	*Attentiveness*	0.603		0.610		
*14*	*Accurate empathy*	0.651		0.667		
15	Collaboration	0.849		0.877		
16	Eliciting automatic thoughts	0.618			0.809	
18	Eliciting meaning/ understanding/ attributions	0.522			0.577	
*19*	*Addressing key issues*	0.436			0.328	
21	Sharing conceptualization with patient	0.424			0.590	
22	Guided discovery	0.670				0.728
23	Asking for evidence/alternative views	0.546				0.683
24	Use of alternative CBT techniques	0.596				0.676
	Fit indices	CFI = 0.666, RMSEA = 0.122 SRMR = 0.103	CFI = 0.712, RMSEA = 0.115, SRMR = 0.102			

**Table 3:** Summary of complex exploratory factor analysis, standardized (*n* = 333).

**Item no**.	**Item description**	**CBT specific skills**	**Session structure**	**Residual factor**
1	Agenda	0.01	**0.53**	−0.19
3	Bridge from previous visit	−0.13	**0.66**	0.01
4	Inquired about ongoing problem	**0.52**	0.17	−0.15
5	Reviewing previous homework	−0.03	**0.41**	0.06
6	Assigning new homework	0.03	**0.52**	0.01
9	Focus/structure	0.81	−0.02	−0.42
10	Socialization to CBT model	0.35	0.56	−0.01
15	Collaboration	0.80	0.08	−0.39
16	Eliciting automatic thoughts	**0.59**	0.16	0.37
18	Eliciting meaning/ understanding/ attributions	**0.56**	−0.06	0.16
21	Sharing conceptualization with patient	0.01	0.66	0.40
22	Guided discovery	**0.74**	−0.03	0.07
23	Asking for evidence/alternative views	**0.63**	−0.01	0.28
24	Use of alternative CBT techniques	**0.49**	0.21	0.13
	Fit indices	CFI = 0.926, RMSEA = 0.064 SRMR = 0.038		

*Items with factor loadings over 0.40 appear in bold and were included in subsequent analyses*.

#### Predictive Validity

Our findings of poor structural validity and partly poor interrater reliability for the CTACS scale may either mean that the scale lack these properties or that our specific sample of patients, therapists, and raters and our procedures were not able to fully reproduce them. Assuming the latter, we chose to proceed with our intended analysis regarding longitudinal associations with subsequent symptom change. Results indicated no statistically significant time-invariant within-level and no between-level effects of competence ratings on symptoms of anxiety and depression (Table 4). This was true for overall competence ratings as well as for the four subscales, even though it should be noted that a near significant within-person effect was observed for therapy structure for depression. Also, for the abbreviated scales derived from the EFA, no statistically significant effects were found. Allowing for time-varying effects of the CTACS (sub)scales or adding client difficulty as a covariate did not substantially alter the results displayed in Table 4, with one notable exception. For the therapeutic relationship subscale, a statistically significant within-person effect was observed at measurement occasion 3 in which higher competence scores for therapeutic relationship predicted lower subsequent depression scores (b = −1.26, 95%CI: −2.28, −0.237, *p* = 0.016). The associated significant Wald-test (*z* = 7.24, df = 2, *p* = 0.027) indicated that the association of therapeutic relationship with depression scores differed across measurement occasions. As the interrater reliability was low for most of the therapeutic relationship items, we conducted sensitivity analyses to examine whether the association of this subscale with depression scores was different across raters. The significant association at measurement occasion 3 was only recovered in cases evaluated by the expert rater (b = −2.52, 95%CI: −4.99, −0.045, *p*= 0.05), but not in cases evaluated by the student rater (b = −0.122, 95%CI: −1.94, 1.70, *p* = 0.90). It should be noted that excluding the fixed therapist effects from the model did not alter the overall results presented above.

**Table 4:** Multilevel associations between CTACS and treatment outcomes in PMHC.

**GAD-7**	**Within-person effect Est. (95% CI)**	***p*-value**	**Between-person effect Est. (95% CI)**	***p*-value**
Overall competence	0.063 (−0.354, 0.479)	0.769	0.687 (−0.283, 1.656)	0.165
Therapy structure	0.032 (−0.399, 0.463)	0.886	0.359 (−0.601, 1.319)	0.464
Therapeutic relationship	0.160 (−0.288, 0.608)	0.484	0.743 (−0.096, 1.583)	0.083
Case conceptualization	0.114 (−0.139, 0.368)	0.377	0.299 (−0.288, 0.886)	0.318
CBT techniques	−0.078 (−0.317, 0.162)	0.524	0.359 (−0.277, 0.995)	0.269
Competence (abbr.)	0.052 (−0.293, 0.397)	0.767	0.516 (−0.442, 1.474)	0.291
CBT techniques (abbr.)	−0.021 (−0.302, 0.260)	0.882	0.551 (−0.179, 1.282)	0.139
Th. structure (abbr.)	−0.008 (−0.277, 0.260)	0.951	−0.132 (−0.872, 0.607)	0.726
**PHQ-9**				
Overall competence	−0.224 (−0.707, 0.260)	0.365	0.563 (−0.680, 1.805)	0.375
Therapy structure	−0.407 (−0.850, 0.036)	0.072	0.555 (−0.728, 1.838)	0.396
Therapeutic relationship	−0.256 (−0.780, 0.267)	0.337	0.804 (−0.232, 1.841)	0.128
Case conceptualization	0.069 (−0.234, 0.372)	0.655	0.156 (−0.605, 0.917)	0.687
CBT techniques	−0.028 (−0.257, 0.202)	0.813	0.007 (−0.806, 0.821)	0.986
Competence (abbr.)	−0.081 (−0.449, 0.288)	0.668	0.106 (−1.096, 1.308)	0.862
CBT techniques (abbr.)	−0.015 (−0.298, 0.268)	0.917	0.169 (−0.743, 1.082)	0.716
Th. structure (abbr.)	−0.164 (−0.443, 0.114)	0.248	−0.183 (−1.039, 0.672)	0.675

## Discussion

We examined the psychometric properties of the Norwegian version of CTACS and its associations with treatment outcome in a sample of primary care clients who received CBT for symptoms of anxiety and/or depression. Summarizing our results, no evidence for divergent validity between the two constructs of competence and adherence were found as indicated by the very high correlation between these two subscales in CTACS (0.97). Regarding reliability, ICCs for the mean score of the full competence scale and its associated subscales were generally good to excellent, although the subscale measuring the quality of the therapeutic relationship was relatively low. Internal consistency for the total scale and most subscales was satisfactory, except for the subscale case conceptualization. Yet, our CFA model results did not provide an acceptable model fit for neither a one-factor nor a four-factor solution. From an EFA including only items (*n* = 14 of 22) with satisfactory interrater reliability, we identified two meaningful factors consisting of CBT specific skills and Session structure. Finally, our results indicated overall no evidence for the scales' predictive validity, with one notable exception. For the therapeutic relationship subscale, at measurement occasion 3 when rated by the expert rater, higher competence scores predicted lower subsequent depression scores.

### Psychometric Properties of CTACS

The lack of divergent validity of the constructs adherence and competence in this study (*r* = 0.97) is a replication of what Barber et al. (2003) reported in their study (*r* = 0.96). Like in Barber et al. (2003), we placed special emphasis on this distinction in the training of raters. Despite this, it seems to have been difficult to differentiate the two. This contradicts previous findings of the two concepts as relatively low associated (Miller and Binder, 2002). At the same time, there is some empirical evidence suggesting that adherence and competence are correlated when the same raters and same items are used (Barber and Critis-Christoph, 1996; Barber et al., 2006; Bjaastad et al., 2016; Haug et al., 2016), albeit not as high as for our and Barber et al.'s findings. It is also conceivable that the problem is related to the design of the scale itself. There is relatively little difference between the question formulation of adherence and competence, and this may have contributed to the high overlap. As suggested by others before (Waltz et al., 1993; Bjaastad et al., 2016), adherence is a prerequisite for competence and another reason for the overlap might thus be that adherent therapists are more competent.

We obtained an acceptable inter-rater agreement for the total and most of the subscales in the CTACS, except for the alliance subscale. Looking at the item level, empathy, warmth, respect, and attentiveness showed the lowest reliability (<0.40). Similar difficulties with a reliable assessment of alliance items were also reported by Barber et al. (2003) and were excluded from the final scale in that study. There may be several explanations for the less reliable judgment of the alliance items in the current study. First, it is plausible that there is a need for more training related to alliance aspects to achieve a satisfactory level of agreement. Albeit, even if more training had improved reliability for the alliance part in our study, it does not guarantee interrater agreement between studies, which possibly could in part explain the observed heterogeneity *between* alliance studies (Flückiger et al., 2018). Second and relatedly, it might be more difficult for novice raters to evaluate alliance and the more abstract items such as case conceptualization than concrete items like setting agenda. However, since Barber et al. only used expert raters and found similar difficulties with alliance items, it might also be reasonable to consider that the alliance items are inherently problematic and need further clarification. Third, the alliance items tended to have less variance than the other items, making it more difficult to achieve covariation between raters. Fourth, the use of audiotaped sessions in both studies, and hence lack of visual cues from non-verbal communication, may have made the alliance items harder to evaluate and hence more unreliable. Finally, and overarching, the qualitative and subjective nature of the alliance may make it harder for third parties to grasp. That said, using observer ratings is a way to avoid other potential biases, such as pleasing behavior or effects related to treatment gain or lack thereof.

Although internal consistency was overall acceptable, it is equally important to understand the structural validity of CTACS. We neither found a one-factor nor a four-factor model structure to fit well with the data in a CFA. Our EFA results were also relatively difficult to interpret for all the investigated factor structures. Based on our overall assessment the most interpretable structure was with three factors, but with only two meaningful factors representing CBT specific skills and Session structure. Perhaps the alliance factor in CTACS would have been clearer if our interrater reliability had been better. As mentioned in the introduction we are not aware of any other attempt to validate the factor structure of CTACS, except for Bjaastad et al. (2016) revised version for youth anxiety disorders revealing a somewhat similar two-factor solution (CBT structure and session goals, and process and relational skills). The lack of a clear structure for CTACS in our study makes it difficult to interpret what the underlying factors measure and demonstrates poor construct validity.

### Associations With Outcome

Overall, our lack of relationship between competence and outcome is in line with a meta-analysis by Webb et al. (2010). However, our findings contradict other meta-analyses suggesting a small competence effect for various disorders in CBT studies (Zarafonitis-Muller et al., 2014) and the positive relationship found between alliance and treatment outcomes regardless of treatment type or diagnosis and at the within-person level early in treatment (Flückiger et al., 2018, 2020). For depression at the between-person level, our results are also not in line with meta-analyses similarly indicating a positive relationship between competence and outcome (Webb et al., 2010; Zarafonitis-Muller et al., 2014). Regarding the latter, our near significant within-level association between CBT therapy structure and depression symptoms might notably be interpreted as indicating a similar trend. Additionally, we found some evidence supporting an alliance and depression outcome association, but only for the expert rater at time point 3.

The uncertain reliability and construct validity of CTACS in the current study might have contributed to the lack of relationship observed between these processes and outcomes. It is also plausible that the effect of competence on outcome might be relatively small and that we were not able to detect these effects in our sample. This potential lack of statistical power was amplified by the clustered nature of observations at the individual and therapeutic level, which resulted in a lower effective sample size than observed. Furthermore, it may be relevant to consider the relatively mild nature of mental health problems in our sample as therapist competence may be more important in clients with severe and complex problems (Johns et al., 2019). Restriction of range may also have impacted the size of the reported correlations although range and variance estimates of the competence scores do not seem to be much lower as compared to other studies using CTACS (Barber et al., 2003; Bjaastad et al., 2016).

Regarding the prediction of alliance-outcome, the client-rated alliance has substantially more empirically support (Castonguay et al., 2006; Horvath et al., 2011) and might be more appropriate to employ than observer ratings. Strikingly, the mentioned meta-analyses report a high degree of heterogeneity and many studies did not separate within- and between-level effects in their analyses that might have introduced bias in estimates. These aspects may all limit comparability to our results. Furthermore, the meta-analysis for CBT competence only consisted of seven competence studies with small sample sizes (*n* = 30–69) and results need therefore to be interpreted with caution.

### Future Research

Although our study points to several psychometric problems of the CTACS, more research is needed before conclusions regarding its utility are drawn. It would be interesting to see whether the lack of structural validity can be reproduced in other samples. It might also be informative to conduct studies where different raters assess adherence and competence of the CTACS to further explore to what extent the high overlap is a consequence of using the same raters for both (Bjaastad et al., 2016). One might reason that using the CTS would be preferable due to it being shorter, highly more used and evaluated (Muse and McManus, 2013). However, this scale also has some ambiguity regarding its psychometric properties and evidence for its predictive validity is thus far limited (Barber et al., 2003; Muse and McManus, 2013; Goldberg et al., 2020). Competence scales' predictive validity is crucial to investigate since the overarching goal for providing competent CBT is to help clients reduce their symptoms (Muse and McManus, 2013). It would be of interest to include both the CTACS and the CTS in one study for further comparison. Another suggestion is to develop new scales (Muse and McManus, 2013) based on modern data analytic and measurement methodologies (Goldberg et al., 2020). We would not necessarily advocate for the development of a new scale, but rather revise the existing version of the CTACS. Key problems areas according to our study were interrater reliability of the alliance items, lack of discriminant validity between adherence and competence, and lack of structural validity. The key to improve these weaknesses would be a thorough review of both response category content and item content. The former is relevant for improving interrater reliability and discriminant validity. The response categories for the alliance items could be made more concrete allowing less room for interpretation. The response categories for the adherence items should also made more concrete and descriptive in nature. Moreover, it should be reviewed whether the distinction between adherence and competence is theoretical and practically meaningful for all items or only for a selection of items. Item content should be revised with structural validity in mind. Items belonging to one sub-dimension should be sufficiently different from other sub-dimensions. Error correlations between items induced due to similar wording should be avoided and vague items should be reformulated. We would also advice to merge data from all previous studies that have used CTACS as this would provide a broader basis to guide and inform a revised version of the scale. In addition to improving measurement properties, a good study design and analysis plan are required to provide more definite answers on the impact of these constructs on outcomes in psychotherapy (i.e. sufficient statistical power, SEM approach in which both measurement error and nested structures are accounted for, repeated measures, multiple raters etc.). According to Cuijpers et al. (2019), the psychotherapy research field has been in the pilot phase for several decades and we need to realize that examining *how* therapy works are completely different from examining *whether* it works. Researchers should preferably aim for amongst others large enough sample sizes, high-quality measures and, investigate temporal associations and separating within- and between-level effects (Cuijpers et al., 2019).

### Strengths and Limitations

The main strengths of the present study are that we follow recommendations from the literature by making a thorough investigation of psychometric properties of the CTACS, having a sound sample size and separate within- and between-level effects in the analysis of associations with outcome (Cuijpers et al., 2019). By conducting the study in a naturalistic setting, we try to maximize the external validity.

We also note some limitations of the current study in addition to the psychometric issues mentioned above. First, we did not have a good enough measure of the alliance in our study as indicated by the poor interrater reliability and structural validity. This might have introduced bias in our results for both psychometric and outcome associations. Nonetheless, since we were able to detect this bias, separate MLM analyses with abbreviated versions in addition to the original full scale and domains were conducted, thereby limiting this potential bias for the CBT specific domains. Still, this is a limitation for our alliance results. Second, our use of session recordings drawn from a primary care context with a relatively heterogenic group of clients is likely both a strength and limitation. It might have increased the generalizability of our associations with outcome results and at the same time, it could potentially have made it more difficult to detect a clear structure in our factor analyses of CTACS. Third, we evaluated only three time points, limiting our ability for investigating the suggested curve-linear relationship between competence and outcome (Barber et al., 2006).

## Conclusion

This study investigated the psychometric properties of the Norwegian version of CTACS and its associations with treatment outcomes in a sample of primary care clients who received CBT for anxiety and/or depression. Psychometric problems were identified regarding interrater reliability and divergent, structural and predictive validity. These discouraging findings for some of the psychometric qualities of the scale might have contributed to the lack of overall associations with outcome in our study, but could also point to limited clinical utility of the scale. We highlighted the importance of providing empirical evidence for both reliability and validity aspects of scales and separating within and between-person effects in analyses of associations with outcome. This is especially important for gaining more confidence in our knowledge of competence and alliance, and hence, what might contribute to the effects of CBT and therapy in general.

## Data Availability Statement

The datasets generated and/or analyzed during the current study are not publicly available due to ethical restrictions and personal data protection but are available from the corresponding author on reasonable request. Requests to access the datasets should be directed to Linn Vathne Lervik, linnvathne.lervik@fhi.no, Robert Smith, robert.smith@fhi.no.

## Ethics Statement

The study was approved by the Regional Ethics Committee for Western Norway, which falls under the Norwegian Ministry of education and research (REK-vest no. 2015/885). All participants have signed an informed consent scheme. The patients/participants provided their written informed consent to participate in this study.

## Author Contributions

All authors designed the study. ORS and LL analyzed the data. LL drafted the manuscript. All authors contributed in interpretation of the data, critical revisions of the draft and read and approved the final manuscript.

## Conflict of Interest

The authors declare that the research was conducted in the absence of any commercial or financial relationships that could be construed as a potential conflict of interest.

## Abbreviations

BIC: Bayesian information criteria
CBT: Cognitive behavioral therapy
CFA: Confirmatory factor analysis
CFI: Comparative fit index
CTACS: Cognitive therapy adherence and competence scale
CTS: Cognitive therapy scale
EFA: Exploratory factor analysis
ES: Effect size
GAD-7: Generalized anxiety disorder 7-item scale
ICC: Intraclass correlation
IAPT: Improving access to psychological therapies
IQR: Inter quartile range
PHQ-9: Patient health questionnaire-9
PMHC: Prompt mental health care
RCT: Randomized control trial
RMSEA: Root mean square error of approximation
SD: Standard deviation
SRMR: Standardized root mean square residual.
