# Supplementary material for: Psychometric Properties of the Norwegian Version of the Cognitive Therapy Adherence and Competence Scale (CTACS) and Its Associations With Outcomes Following Treatment in IAPT Norway
Source: Front Psychol. 2021 Feb 16;12:639225. doi: 10.3389/fpsyg.2021.639225 (PMC7921157; doi:10.3389/fpsyg.2021.639225)

Supplementary information

Scatterplot of standardized residuals of competence score accounting for correlated observations across time and modelling the therapist variable as a fixed effect. After partialling out therapist effects, significant variance in competence scores remain across all time points.

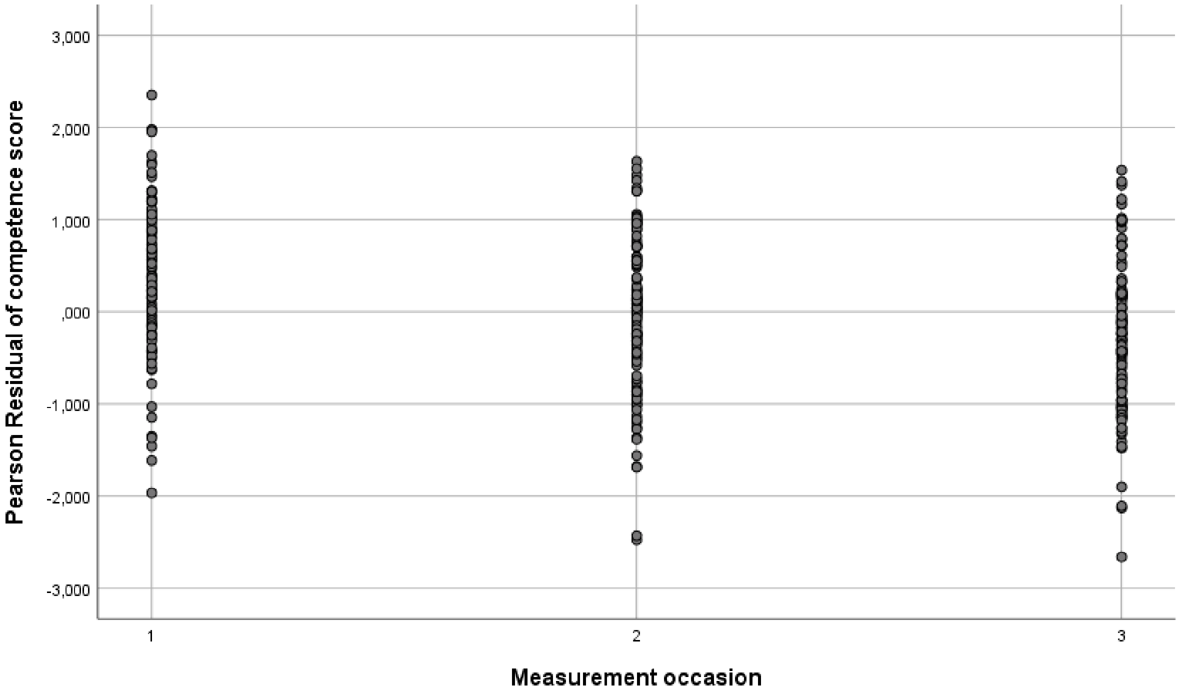

Supplement: Supplementary file 1 [file Data_Sheet_1.pdf]
